# Analysis of the ethanol stress response mechanism in *Wickerhamomyces anomalus* based on transcriptomics and metabolomics approaches

**DOI:** 10.1186/s12866-022-02691-y

**Published:** 2022-11-15

**Authors:** Yinfeng Li, Hua Long, Guilan Jiang, Xun Gong, Zhihai Yu, Mingzheng Huang, Tianbing Guan, Yuanyuan Guan, Xiaozhu Liu

**Affiliations:** 1 Guizhou Institute of Technology, Guiyang, 550000 People’s Republic of China; 2 Chongqing University of Science and Technology, Chongqing, 404100 People’s Republic of China; 3 Henan Institute of Science and Technology, Xinxiang, 453000 People’s Republic of China

**Keywords:** Ethanol stress, Growth, W. anomalus, Transcriptomics, Metabolomics

## Abstract

**Background:**

*Wickerhamomyces anomalus* (*W. anomalus*) is a kind of non-*Saccharomyces* yeast that has a variety of unique physiological characteristics and metabolic features and is widely used in many fields, such as food preservation, biomass energy, and aquaculture feed protein production. However, the mechanism of *W. anomalus* response to ethanol stress is still unclear, which greatly limits its application in the production of ethanol beverages and ethanol fuels. Therefore, we checked the effects of ethanol stress on the morphology, the growth, and differentially expressed genes (DEGs) and metabolites (DEMs) of *W. anomalus*.

**Results:**

High concentrations of ethanol (9% ethanol and 12% ethanol) remarkably inhibited the growth of *W. anomalus*. Energy metabolism, amino acid metabolism, fatty acids metabolism, and nucleic acid metabolism were significantly influenced when exposing to 9% ethanol and 12% ethanolstress, which maybe universal for *W. anomalus* to response to different concentrations of ethanol stressl Furthermore, extracellular addition of aspartate, glutamate, and arginine significantly abated ethanol damage and improved the survival rate of *W. anomalus.*

**Conclusions:**

The results obtained in this study provide insights into the mechanisms involved in *W. anomalus* response to ethanol stress. Therefore, new strategies can be realized to improve the ethanol tolerance of *W. anomalus* through metabolic engineering.

**Supplementary Information:**

The online version contains supplementary material available at 10.1186/s12866-022-02691-y.

## Introduction

*Wickerhamomyces anomalus* (*W. anomalus*), formerly known as *Pichia anomala*, *Hansenula anomala*, and *Candida pelliculosa*, possesses a variety of unique physiological characteristics and metabolic features [1–3], such as tolerance to a wide range of extreme environmental conditions [4]. *W. anomalus* secrete many kinds of glycosidases to improve the flavor of foods, generating numerous volatile ester compounds with floral and fruity aromas, and these compounds are safe for people and livestock [5, 6]. *W. anomalus* has been widely applied in various industrial fields including in food preservation and processing [7], the energy and chemical industry [8], and aquaculture feed protein production [9]. However, the mechanism of the yeast cell response to ethanol stress has not been fully elucidated, which greatly limits its application in the production of ethanol beverages and ethanol fuels.

Studies have shown ethanol stress can trigger changes in a large number of genes and signal transduction pathways that are related to cell structure, substance metabolism and transport, and stress response [10, 11]. However, the underlining mechanism of the response to ethanol stress may be different for different yeast species. For example, *Saccharomyces cerevisiae* improved the stability of cell membrane proteins and the cell membrane by increasing the synthesis of ergosterol and proline transporters under ethanol stress [12, 13]. In contrast, *Kluyveromyces marxianus* stabilized its cell membrane structure by reducing the expression of some genes related to ergosterol and fatty acid synthesis [14, 15]. For *W. anomalus*, the specific genes and pathways influenced by ethanol stress remain unclear.

At the metabolic level, yeast cells can cope with ethanol stress by adjusting various intracellular metabolic reactions and the contents of metabolites. For *S. cerevisiae*, the energy demand of yeast cells was maintained by adjusting the intracellular glucose metabolism pathways (glycolysis and TCA cycle), which helped cells to relieve the damage caused by an adverse environment (ethanol stress) [16, 17]. In addition, fatty acid metabolism, amino acid metabolism, trehalose metabolism, and other metabolic pathways were regulated, cell membrane structure and components were reconstructed, endogenous protective substances (such as a variety of amino acids and trehalose) were recruited, the hydrophilic environment around protein molecules changed, and the aggregation and precipitation of proteins were reduced when *S. cerevisiae* encountered ethanol stress [18–20]. Thus, the structural stability of proteins and cells was maintained, and the damage caused by ethanol was reduced. Moreover, carbon metabolism, amino acid metabolism, trehalose metabolism, and ergosterol metabolism are known to be involved in the process of responding to ethanol stress for *K. marxianus* [21]. The metabolites involved in the response of different yeast species to ethanol stress may also be different. For example, glutamate was confirmed to be involved in the *K. marxianus* stress response to ethanol, but not in the *S. cerevisiae* response. Therefore, it is necessary to explore the metabolites of *W. anomalus* in response to ethanol stress.

In our previous study, an intense aroma-producing yeast strain, *W. anomalus* C11, was obtained from the spontaneous fermentation of *Rosa roxburghii* Tratt by the sniffing method [22]. This strain yeast tolerated 9% (v/v) ethanol treatment. In the present study, we combined RNA-seq and liquid chromatography technologies to explore the ethanol stress response and ethanol tolerance mechanisms of *W. anomalus*. To the best of our knowledge, this study represents the first integrated transcriptomic and metabolomics analysis to reveal the ethanol stress response mechanism of *W. anomalus*.

## Results

### Effects of ethanol stress on morphological characteristics of W. anomalus

**Fig. 1:**
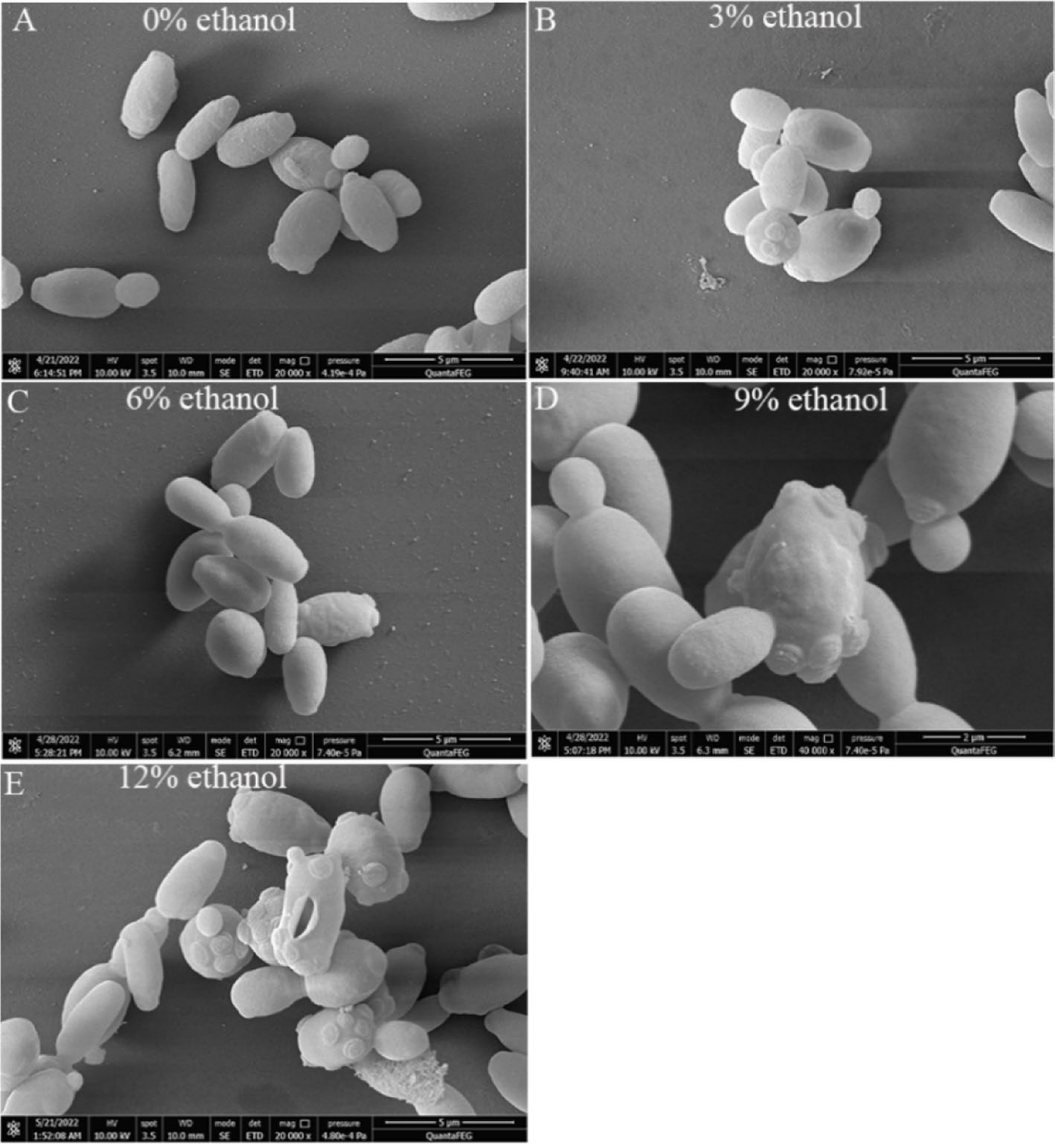
Morphological characteristics of *W. anomalus* under different ethanol treatments. **A** control (0% ethanol); **B** 3% ethanol treatment group; **C** 6% ethanol treatment group; **D** 9% ethanol treatment group; E) 12% ethanol treatment group. Bar = 5 μm

As shown in Fig. 1A, W*. anomalus* cells were ellipsoidal, full, and smooth when no ethanol stress was induced (0% ethanol group). With the increase in ethanol concentration, the fullness decreased and the surface became uneven and deformed in many *W. anomalus* cells (Fig. 1B to 1D). The surface cracks and ruptured cells appeared when the ethanol concentration increased to 12% (Fig. 1E).

### Effects of ethanol stress on the growth of W. anomalus

**Fig. 2:**
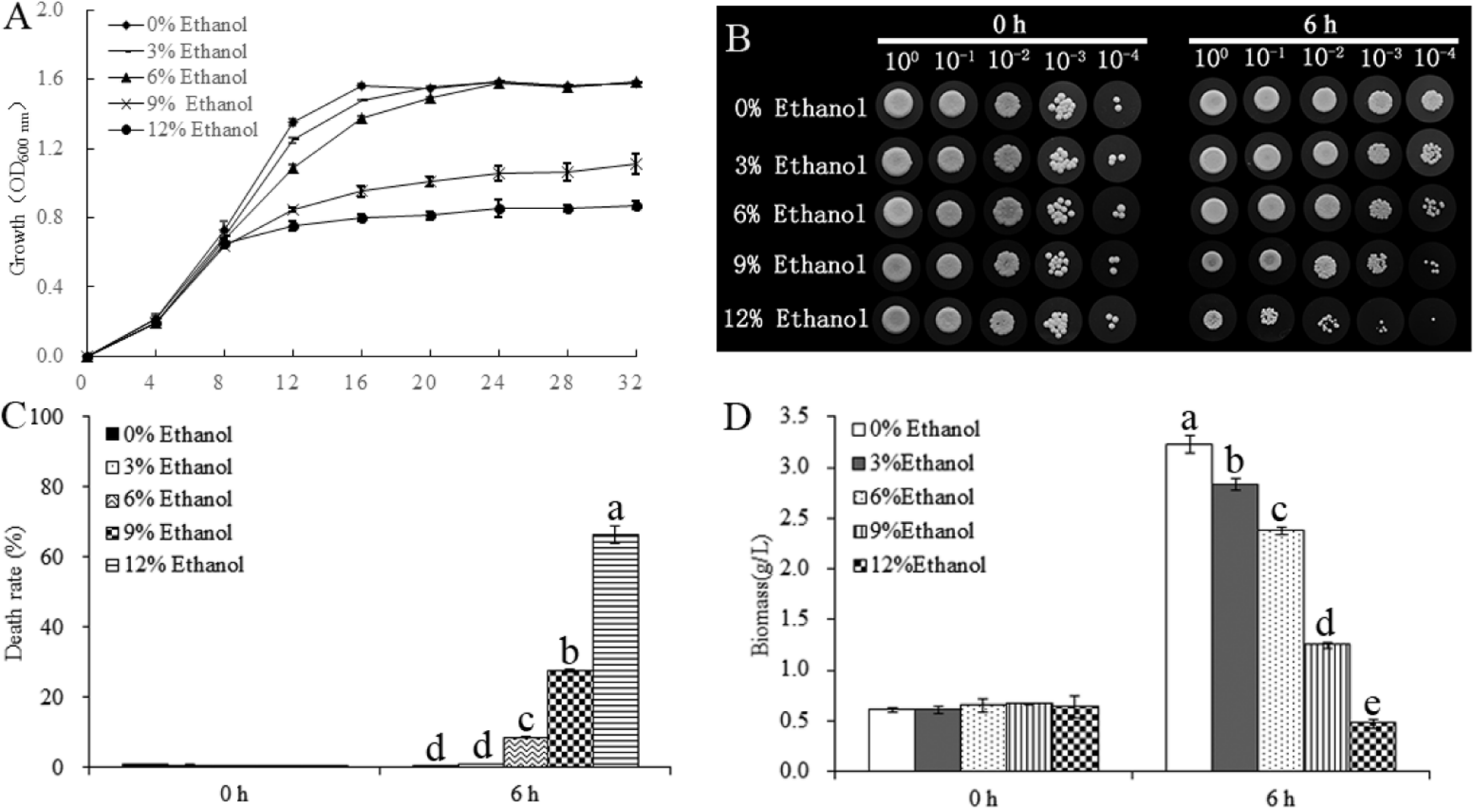
Effects of different ethanol treatments (0%, 3%, 6%, 9%, and 12% v/v) on the growth of *W. anomalus*. **A** Growth curves of *W. anomalus*; **B** Survival rate of *W. anomalus*; **C** Death rate of *W. anomalus*; **D** Biomass of *W. anomalus* after ethanol treatment. Values in the same column with different lowercase letters are significantly different (*P* < 0.05)

The effects of ethanol stress on the growth of *W. anomalus* are exhibited in Fig. 2A. The 3% and 6% ethanol treatments mainly inhibited the growth of *W. anomalus* cells in the logarithmic growth phase, prolonging it, but these treatments had little effect on the yeast cells during the stationary phase. The 9% and 12% ethanol treatments strongly inhibited the growth of *W. anomalus*, shortening the logarithmic growth period and causing the yeast cells to quickly enter the stationary phase.

The effects of ethanol stress on the survival of *W. anomalus* were monitored and depicted in Figs. 2B and 2C. Yeast growth of all the groups was similar before the stress treatment (0 h). The survival rate of yeast cells in the 3% and 6% ethanol treatment groups was lower than that of the control group (0% ethanol) after 6 h of ethanol treatment. The survival rate of the 9% ethanol treatment group was significantly lower than that of the control. Most of the cells in the 12% ethanol treatment group died, and the survival rate was much lower than in the control group. In the 3% and 6% ethanol treatment groups, 1%and 8.6% of cells were stained blue, respectively, indicating a low number of dead cells. In contrast, 27.54% and 66.33% of the cells were stained blue in the 9% and 12% ethanol treated groups, respectively (Fig. 2C, Fig. S1). Therefore, the survival of *W. anomalus* decreased with increasing ethanol concentration.

In addition, ethanol stress also reduced the biomass of *W. anomalus*, and the biomass value gradually decreased with increasing ethanol concentration. Among these treated groups, 12% ethanol treatment had the strongest inhibited impact on the biomass of *W. anomalus*, while the 3% ethanol treatment had the weakest inhibited impact (Fig. 2D). Collectively, a high concentration of ethanol (9% ethanol and 12% ethanol) remarkably inhibited the growth (survival rate and death rate) of *W. anomalus*. Therefore, 9% and 12% ethanol were selected as the treatment concentrations for gene expression profile (transcriptomics) and metabolic profile analysis (metabolomics).

### Effects of ethanol stress on the expression of genes in W. anomalus

Here, 7,141,520,437, 6,897,852,543, and 6,664,489,895 raw bases were obtained in 0%, 9%, and 12% ethanol treatment groups, respectively. The 0%, 9%, and 12% ethanol treatment groups obtained 6,969,933,135, 6,735,048,208 and 6,501,374,566 clean bases after data processing, respectively. The error rate in these three groups was 0.02%, and the values of Q20 (%) and Q30 (%) were greater than 90% (Table 1). In addition, principal component analysis (PCA) results indicated each group was in a separate confidence circle (Fig. S2). The data suggest the sample quality was acceptable, and the sequencing data were accurate for this transcriptome sequencing.

**Table 1:** Quality analysis of transcriptome sequencing data of *W. anomalus* with ethanol stress

Group	Raw reads	Raw bases	Clean reads	Clean bases	Error rate (%)	Q20 (%)	Q30 (%)	GC/%
0% Ethanol	47,294,837.33	7,141,520,437	46,859,291.33	6,969,933,135	0.02	98.75	95.84	38.65
9% Ethanol	45,681,142.67	6,897,852,543	45,300,448.67	6,735,048,208	0.02	98.70	95.70	41.07
12% Ethanol	44,135,694.67	6,664,489,895	43,675,678	6,501,374,566	0.02	98.12	94.13	38.19

**Fig. 3:**
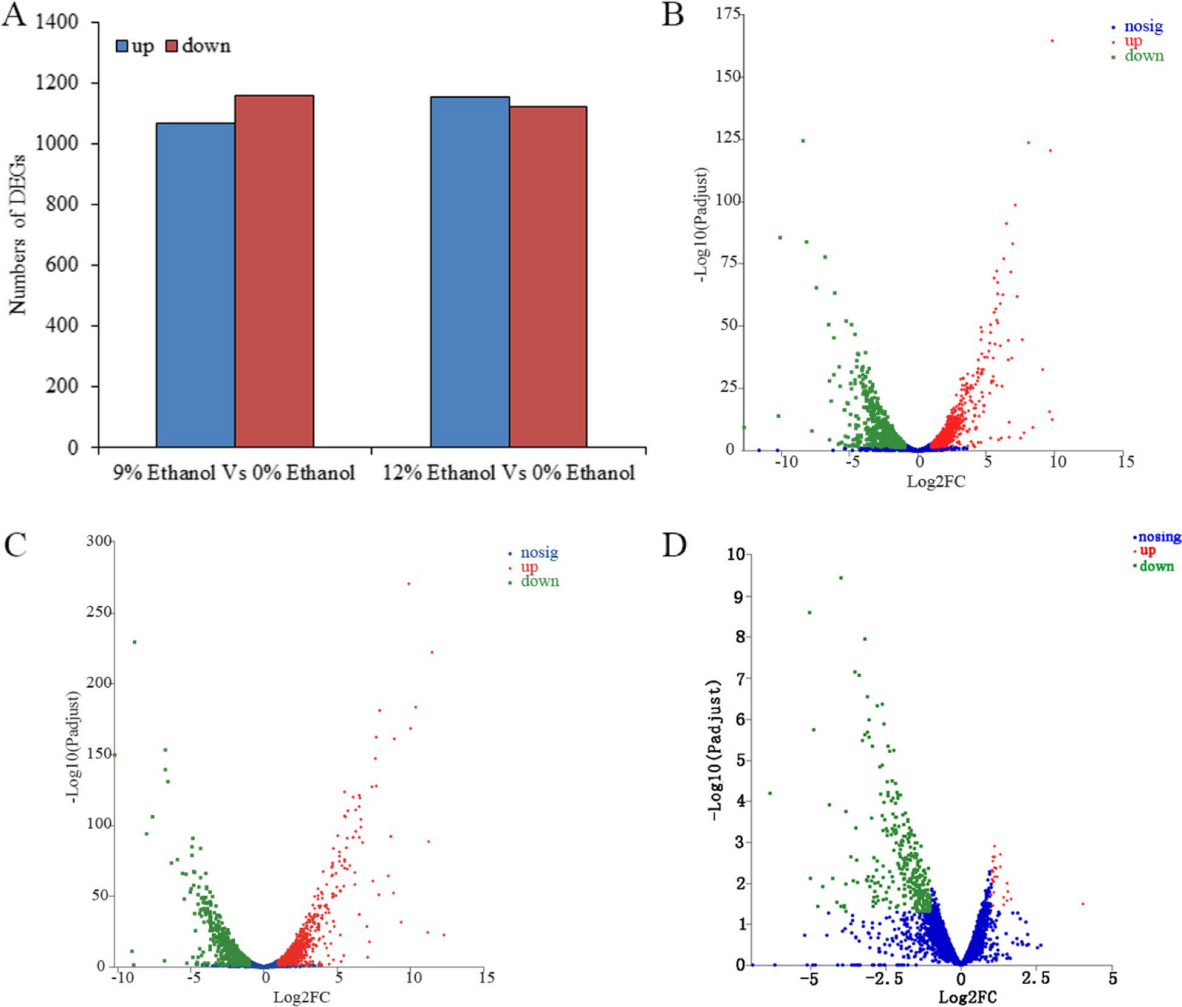
Differentially expressed genes (DEGs) of *W. anomalus* under ethanol stress. **A** Bar chart of DEGs; **B** Volcano plot of DEGs between the 9% ethanol treatment group and the control group; **C** Volcano plot of DEGs between the 12% ethanol treatment group and the control group; **D** Volcano plot of DEGs between the 12% ethanol treatment group and 9% ethanol treatment group

To identify the differentially expressed genes (DEGs) of *W. anomalus* with ethanol stress, we compared gene expression levels among the ethanol-treated groups and the control group, with a filter condition set to *P* values < 0.05 and | log2 (FC) |> 2. There were 2227 DEGs found in the 9% ethanol treatment group compared to control group, of which 1,068 DEGs were upregulated and 1159 DEGs were downregulated (Figs. 3A and B, Table S1). In addition, 2274 DEGs were common between the 12% ethanol treatment group and the control group, of which 1153 DEGs were upregulated and 1121 DEGs were downregulated (Figs. 3A and C, Table S2). In addition, 345 DEGs were detected between 12 and 9% ethanol treated groups, 25 DEGs were upregulated and 320 DEGs were downregulated (Fig. 3D, Table S3).

**Fig. 4:**
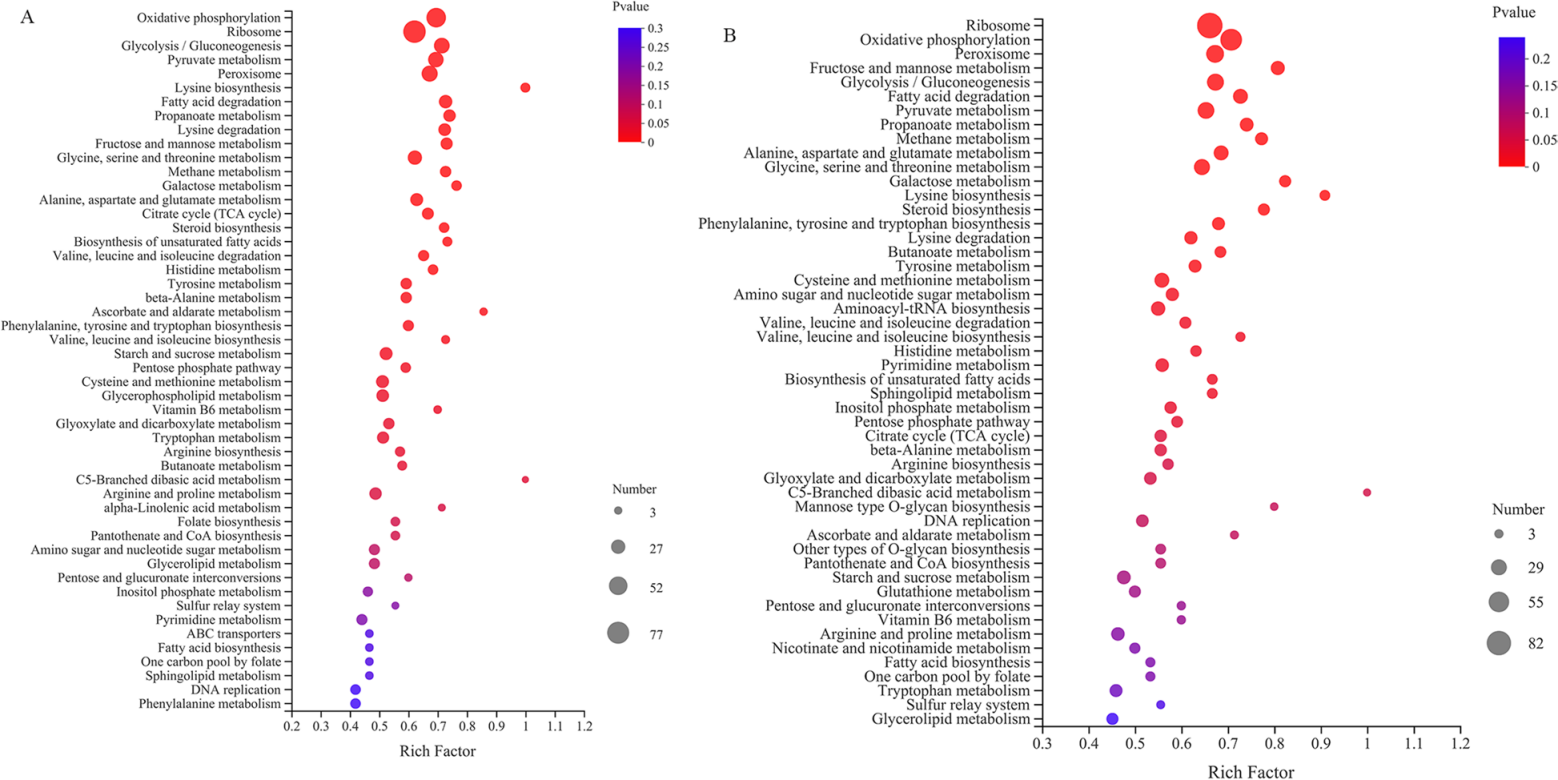
KEGG pathway enrichment of the DEGs of *W. anomalus* under (**A**) 9% and (**B**) 12% ethanol treatments

Moreover, the DEGs induced by ethanol stress were further analyzed via KEGG pathway enrichment, and the data shows the following pathways: oxidative phosphorylation, glycolysis/gluconeogenesis, ribosome, pyruvate metabolism, lysine biosynthesis, propanoate metabolism, fatty acid degradation, glycine, serine, and threonine metabolism, were significantly enriched*.* These pathways are related to energy metabolism (oxidative phosphorylation, glycolysis/gluconeogenesis, fructose, and mannose metabolism), amino acid metabolism (lysine biosynthesis, glycine, serine, and threonine metabolism), protein synthesis (ribosome), fatty acid metabolism (fatty acid degradation, biosynthesis of unsaturated fatty acids, steroid biosynthesis), and oxidative stress regulation (peroxisome) (Fig. 4). In addition, the numbers of enriched DEGs in some enrichment pathways were different between 9% treated group and 12% ethanol treated group. For axample, the number of enriched ribosme in 9% treated group was 77, and which was 82 in 12% treated group (Fig. 4A and B).

### Effects of ethanol stress on the metabolites of W. anomalus

The quality control analysis of the metabolome sequencing samples was carried out by PCA, and the results show sequencing samples of each group in positive and negative ion modes were densely clustered, and the repeatability of samples was stable (Fig. S3).

**Fig. 5:**
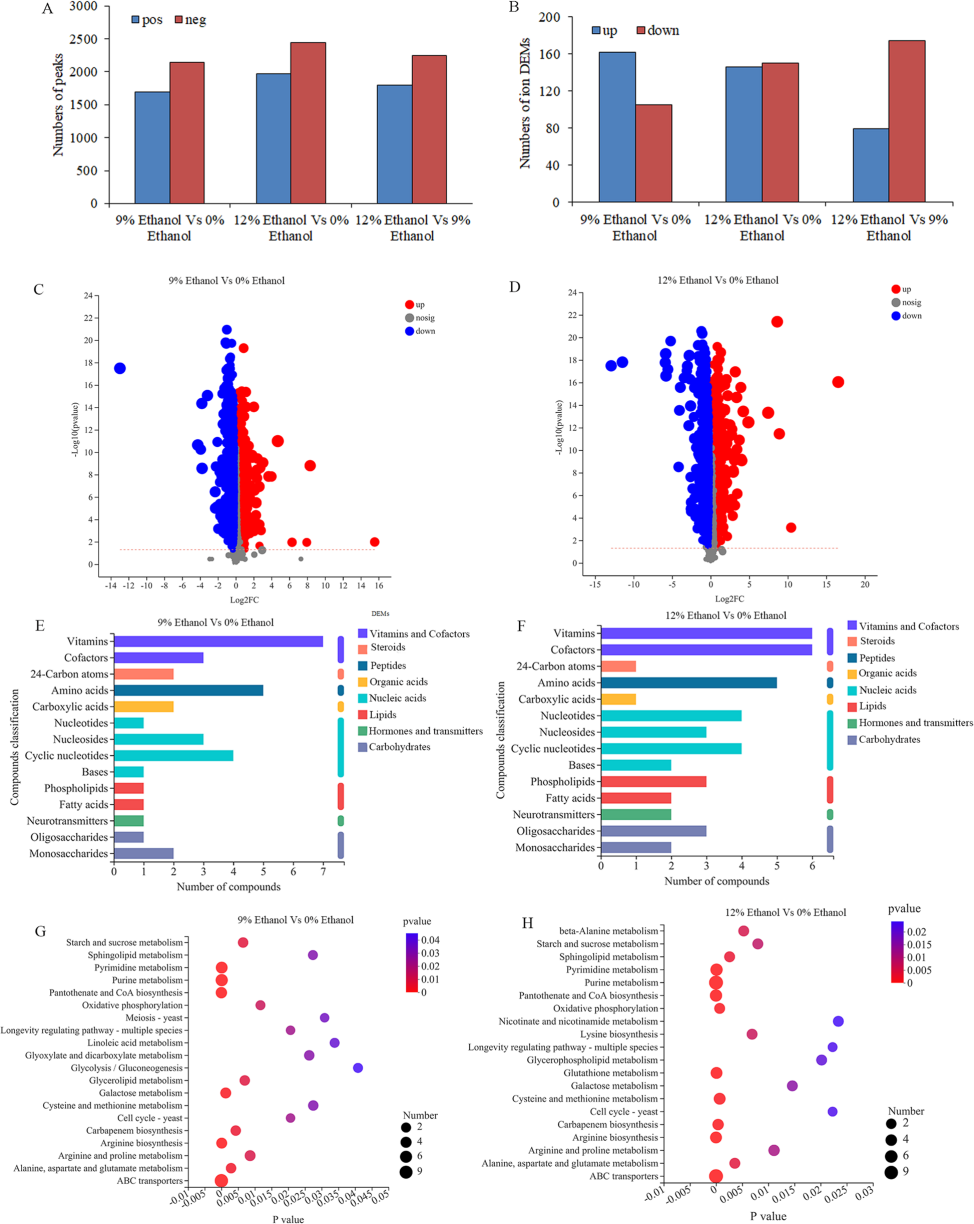
Differentially expressed metabolites (DEMs) of *W. anomalus* induced by ethanol stress. **A**) Comparison of the number of upregulated and downregulated ion peaks detected in positive and negative ion modes for samples treated with different percentages of ethanol; **B**) DEMs induced by 9% and 12% ethanol treatments; **C**) Volcano plot comparing the DEMs between the 9% ethanol treatment group and control group; **D**) Volcano plot comparing the DEMs between the 12% ethanol treatment group and control group; **E**) Identification of DEMs by KEGG database in the 9% ethanol treatment group and control group; F) Identification of DEMs by KEGG database in the 12% ethanol treatment group and control group; G) Identification of enriched DEMs by KEGG database in the 9% ethanol treatment group and control group; G) Identification of enriched DEMs by KEGG database in the 12% ethanol treatment group and control group

Differentially expressed metabolites (DEMs) were screened with the criteria of variable importance in the projection (VIP) > 1 and *P* < 0.05. There were 3845 ion peaks and 267 annotated DEMs detected between the 9% ethanol treatment group and control group in positive and negative ion modes. Among them, 162 DEMs were upregulated and 105 were downregulated (Figs. 5A–C, Table S4). In addition, 4,409 ion peaks and 296 annotated DEMs, including 146 upregulated DEMs and 150 downregulated DEMs, were obtained between the 12% ethanol treatment group and control group (Figs. 5A, 5B, and 5D, Table S5). There were totally 4037 ion peaks and 253 annotated DEMs were found comparing 12% ethanol treated group to 9% ethanol treated group, of which 79 DEMs were upregulated and 174 DEMs were downregulated (Figs. 5A and 5B, Table S6). Furthermore, most of these DEMs were identified as vitamins, amino acids, nucleic acids, and cofactors, by comparing them with the KEGG compound database. However, the identified numbers of cofactors, nucleotides, base, phospholipids and oligosaccharides were higher in 12% ethanol treated group than 9% ethanol treated group (Figs. 5E and F).

We further enriched the DEMs using the KEGG database and found that most DEMs were enriched in the pathways related to yeast growth, such as cell cycle-yeast, longevity regulating pathway-multiple species, and meiosis-yeast (Figs. 5G and H). The following pathways: amino acid metabolism (arginine biosynthesis, alanine, aspartate and glutamate metabolism, arginine and proline metabolism), energy metabolism (oxidative phosphorylation, galactose metabolism, starch and sucrose metabolism), and nucleic acid metabolism (purine metabolism, pyrimidine metabolism), were also enriched. Therefore, we deduced ethanol stress had pleiotropic effects on the metabolic response of *W. anomalus*, including energy metabolism, protein biosynthesis, and nucleic acid metabolism.

### Integrated transcriptomics and metabolomics analyses

**Fig. 6:**
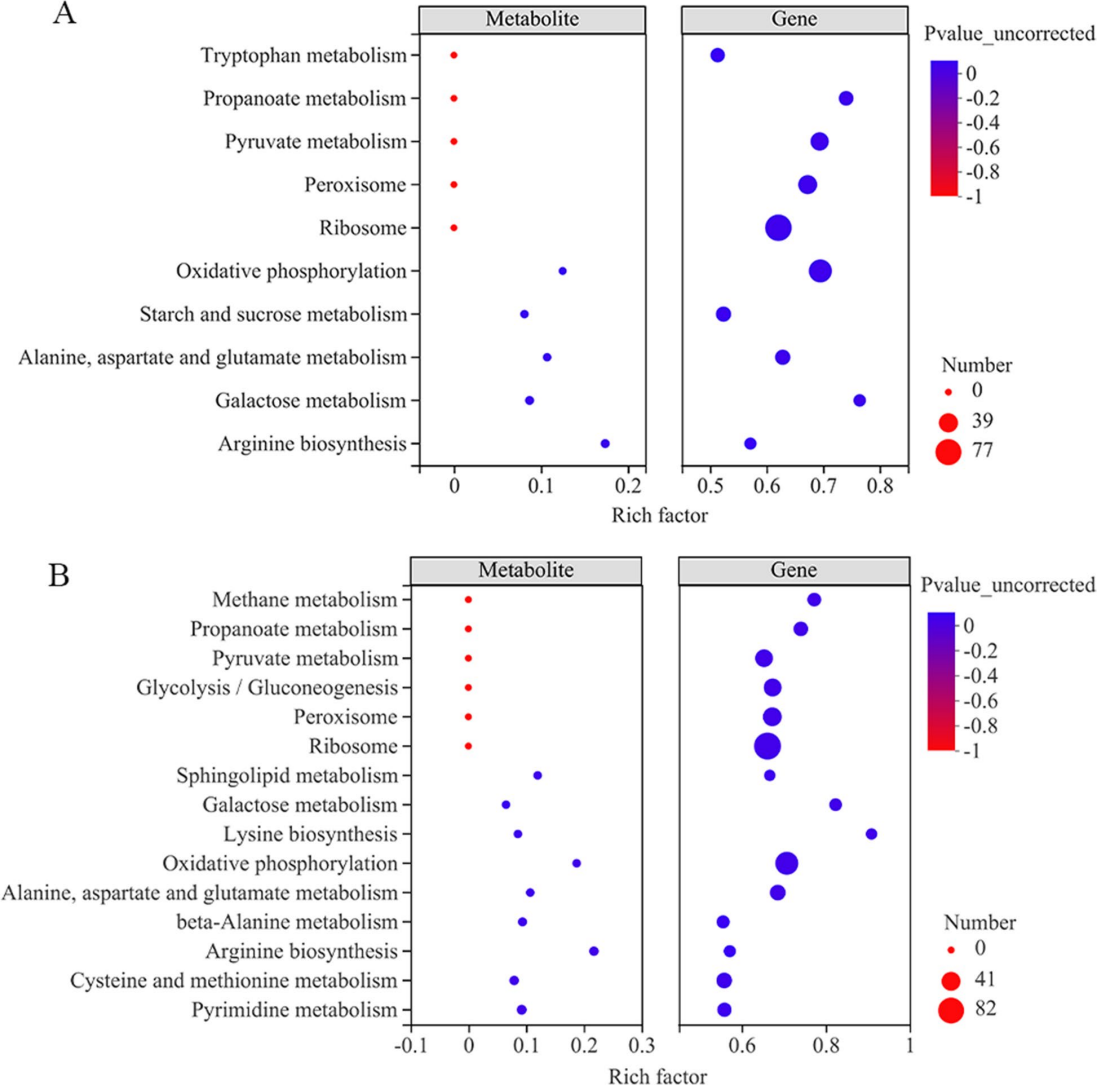
DEGs and DEMs identified in *W. anomalus* under ethanol stress by integrating transcriptomics and metabolomics analyses. **A** DEGs and DEMs between the 9% ethanol treatment group and control group; **B** DEGs and DEMs between the 12% ethanol treatment group and control group

Integrated analysis was performed to further identify the DEGs obtained from transcriptomics and DEMs obtained from metabolomics. The results are shown in Fig. 6. Most of the significantly enriched DEGs and DEMs were associated with oxidative phosphorylation and galactose metabolism. Metabolic pathways, such as aspartate and glutamate metabolism, starch and sucrose metabolism, and arginine biosynthesis, were also enriched. Sphingolipid metabolism, lysine biosynthesis, cystcine and methionine metabolism an pyrimidine metabolism were only enriched in 12% ethanol treatment group. Validation of differentially expressed genes and metabolites.

To quantitatively verify the reliability of the transcriptomics results, the gene expression in five upregulated and five downregulated genes were confirmed by q-PCR. As demonstrated in Fig. 6, *Wa60613*, *Wa78611*, *Wa13980*, *Wa63188,* and *Wa46076* were proved to be upregulated, while *Wa31465*, *Wa92106*, *Wa34481*, *Wa60885,* and *Wa26420* were downregulated (Fig. 6). These results are in good agreement with the transcriptomics data.

**Fig. 7:**
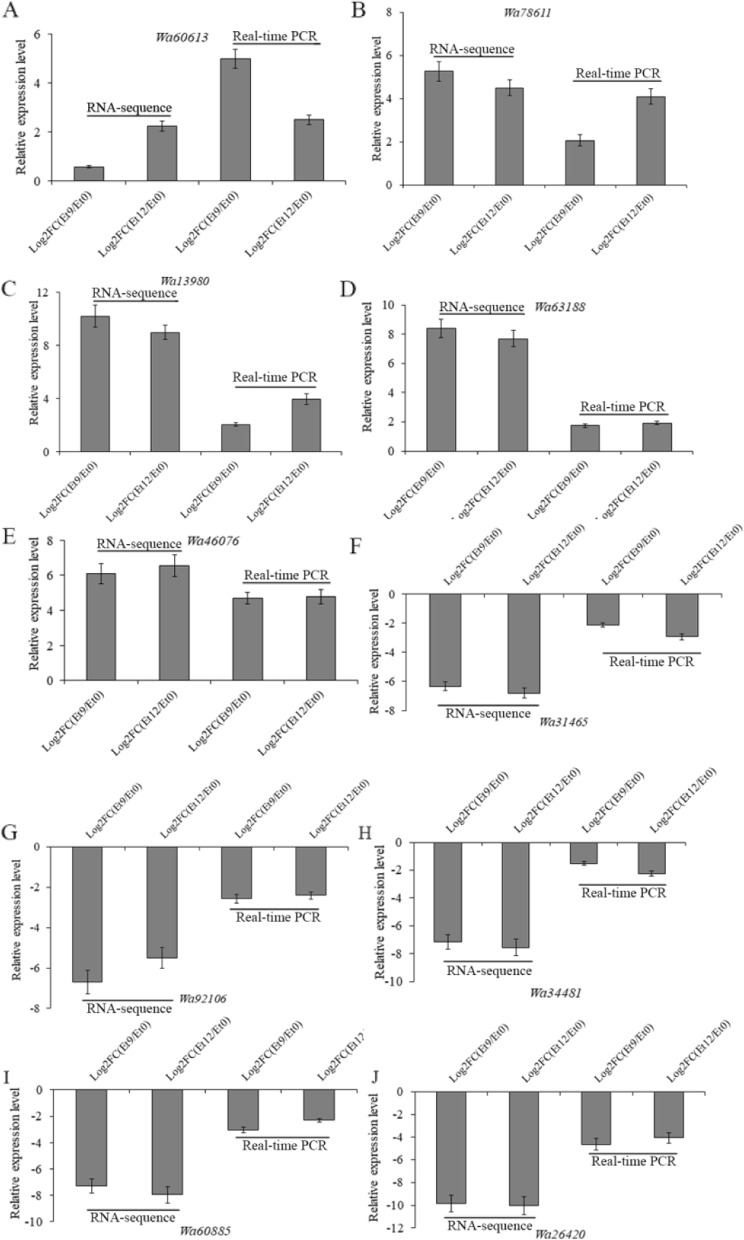
PCR validations of RNA-seq data for genes **A** *Wa60613*; **B** *Wa78611*; **C** *Wa13980*; **D** *Wa63188*; **E** *Wa46076*; **F** *Wa31465*; **G** *Wa92106*; **H** *Wa34481*; **I** *Wa60885*; **J** *Wa26420*

**Fig. 8:**
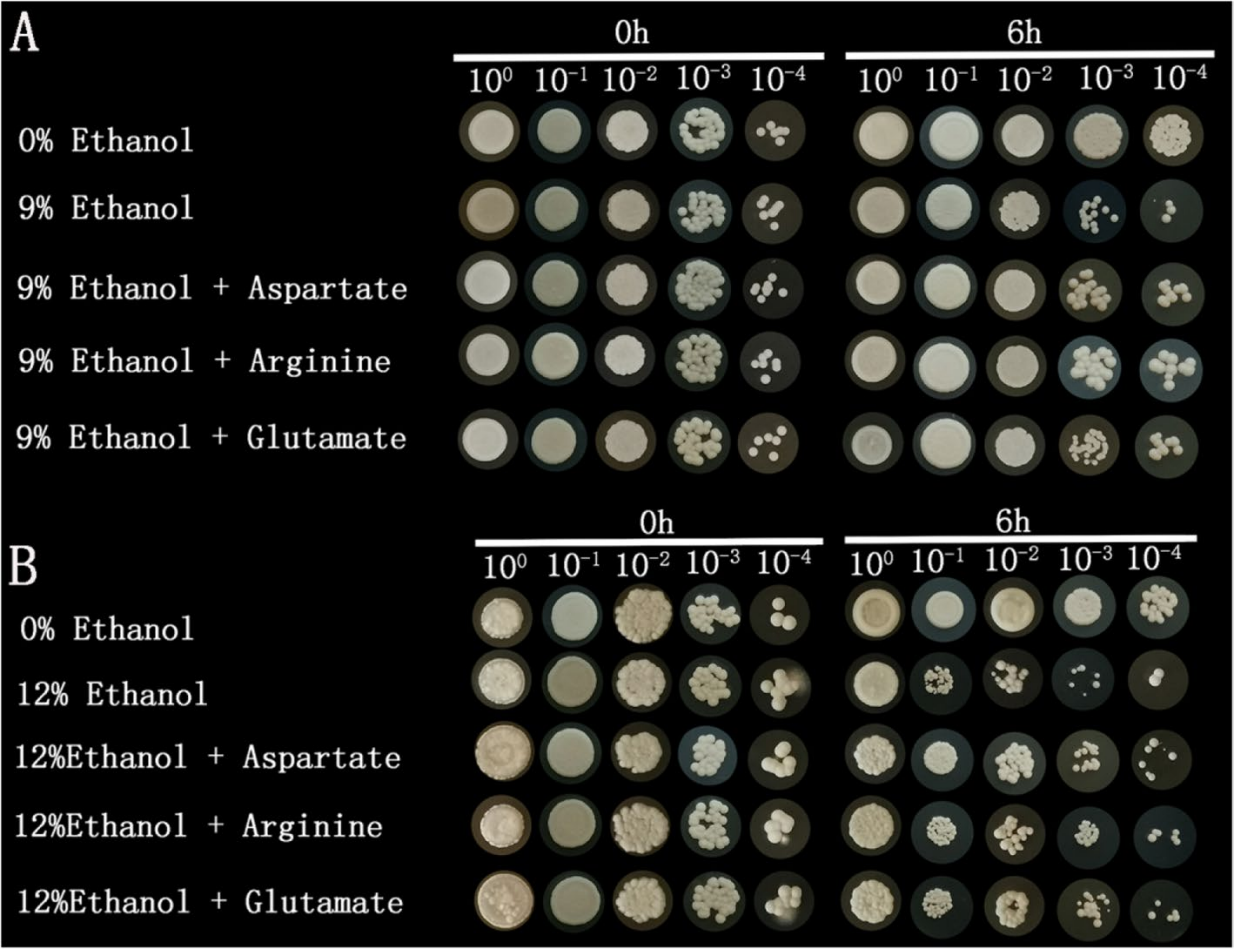
Exogenous supplementation of aspartate, glutamate, and arginine improved the survival rate of *W. anomalus*. **A** Survival of yeast cells in 9% ethanol; **B** Survival of yeast cells in 12% ethanol

The transcriptomics and metabolomics data show aspartate, glutamate, and arginine were involved in regulating the response of *W. anomalus* to ethanol stress (Fig. 7). To further confirm the roles of aspartate, glutamate, and arginine in the response of *W. anomalus* to ethanol stress, the cells were analyzed after the exogenous addition of these amino acids. As demonstrated in Fig. 8, the yeast growth was constant at 0 h under all treatments. However, the yeast growth was significantly inhibited by 9% and 12% ethanol treatments at 6 h. In contrast, the effects of the ethanol treatments were partly mitigated by the addition of exogenous aspartate, glutamate, and arginine, and the cell survival rate of *W. anomalus* increased. Therefore, the extracellular addition of aspartate, glutamate, and arginine can abate the damage due to ethanol stress and improve the survival of *W. anomalus.*

## Discussion

Studies have shown that ethanol stress has pleiotropic effects on yeast cells, including the regulation of gene expression and signal transduction pathways [23]. The response of yeast cells to ethanol stress is complex and involves multiple genes, components, and metabolic pathways. Therefore, the integration of transcriptomics and metabolomics techniques enhances the understanding of the molecular mechanisms and yeast cell response to ethanol stress.

Transcriptomics is a powerful technique that can elucidate gene transcriptional regulation by monitoring the expression profiles of all genes in a certain physiological state. It has been widely used to explore the mechanism of various stress responses in cells [24, 25]. Li et al. identified 937 DEGs in the strain of *S. cerevisiae* Sc131 upon exposure to ethanol stress [12, 26]. Most of the significantly upregulated DEGs were filamentous growth-related genes (such as *OPI1*, *MIT1*, and *GLK1*), mitochondrial energy metabolism-related genes (such as *COX1*, *COX2*, and *COX3*), amino acid metabolism-related genes, as well as protein folding- and protein degradation-related genes. In contrast, ribosome biosynthesis-related genes were significantly downregulated. For *K. marxianus*, the expression of heat shock proteins significantly increased, while the expression of genes related to carbon metabolism, ribosome biosynthesis, unsaturated fatty acid, and sterol biosynthesis remarkably decreased under ethanol treatment. Therefore, energy metabolism, amino acid metabolism, nucleic acid metabolism, and fatty acid metabolism pathways may be the common target of ethanol attack for yeasts, including *S. cerevisiae*, *K. marxianus,* and *W. anomalus* [14]. It is worth noting that the expression of trehalose metabolism-related genes was upregulated at low concentrations of ethanol rather than high concentrations of ethanol in *K. marxianus *[14, 15], but these genes were not found changed in *S. cerevisiae* or *W. anomalus* subjected to ethanol stress.

In a recent study, a metabolomics approach, using gas chromatography coupled with mass spectrometry, was performed to investigate the metabolic changes of *S. cerevisiae* under ethanol stress. The authors found the profiles of fatty acids changed significantly [27]. In a study conducted by Li et al., unsaturated fatty acids played a positive role in the response of *S. cerevisiae* to ethanol stress [28]. In the present study, we also observed fatty acids and fatty acid metabolism-related genes changed dramatically in *W. anomalus.* The functions of fatty acids in the response to ethanol stress can be further confirmed in future research.

Microorganisms have developed different strategies to minimize the adverse effects of ethanol stress, and several of them are connected to amino acid metabolism. For instance, the intracellular accumulation of L-proline protected *S. cerevisiae* from damage caused by ethanol stress, which supports the ethanol tolerances observed in yeast cells [19, 29]. Enrichment analysis also revealed the prominent role of amino acid metabolism in ethanol stress for *W. anomalus* (Fig. 7). Therefore, aspartate, glutamate, and arginine were supplemented to further enhance the survival rate of yeast cells under ethanol stress. It was reported that arginine could protect *S. cerevisiae* cells from ethanol damage [30]. In the present study, we found arginine also played a protective role in *W. anomalus* cells. However, the role of glutamate in ethanol stress may differ for different yeast species. Glutamate was proved to be involved in the ethanol stress response of *K. marxianus* [21] and *W. anomalus* in this study, but it has not been confirmed in *S. cerevisiae*. Glutamate is required for synthesis of protective compound glutathione and indeed the increased content of glutathione was observed during the ethanol stress (data were not shown). But, further investigation of the underlying mechanisms of these amino acids roles in the ethanol response of *W. anomalus* is needed.

## Conclusions

To the best of our knowledge, this was the first study to systematically analyze the effect of ethanol stress on the morphological characteristics and growth of *W. anomalus,* and the response mechanism of ethanol stress, based on transcriptomics and metabolomics approaches. High concentrations of ethanol (9% and 12%, v/v) remarkably inhibited the growth of *W. anomalus*. Energy metabolism, amino acid metabolism, fatty acids metabolism, and nucleic acid metabolism changed significantly in response to ethanol stress. Thus, the results obtained in this study could provide insights into the mechanisms involved in the response of *W. anomalus* to ethanol stress, enabling future metabolic engineering approaches to improve its ethanol tolerance.

## Materials and methods

### Yeast strains and culture conditions

The *W. anomalus* C11 strain was separated from the fruit spontaneous fermentation broth of *R. roxburghii* and cultured with yeast extract peptone dextrose medium (YEPD) at 28 °C for 72 h. The culture was kept at 4 °C for later use.

### Scanning electron microscopy (SEM) analysis

*W. anomalus* cells were cultured with a YEPD medium at 28 °C for 8 h. Different concentrations of ethanol (3%, 6%, 9%, and 12%, v/v) were added, and the cells were cultured for 6 h to implement ethanol stress. Cells cultured without ethanol (0% Ethanol) were used as the control for this experiment and other experiments in this study. Yeast cells were centrifuged at 4, 000 × g for 10 min and washed three times with physiological saline. Then, the cells were re-suspended in 2.5% glutaraldehyde for 4 h at 4 °C and washed three times with 0.1 M PBS (pH 7.4). Subsequently, the cells were eluted with a gradient concentration of ethanol solutions (0%, 50%, 70%, 85%, 95%, and 100%) for 15 min each. Yeast cells were dried with a critical point dryer (Hitachi, Japan), coated with a gold/palladium alloy, and observed with an FEI Quanta FEG 450 scanning electron microscopy system (FEI, USA).

### Growth analysis of W. anomalus under ethanol treatment

For the growth curve determination, *W. anomalus* cells were inoculated into YEPD liquid medium and incubated at 28 °C with 180 rpm shaking for 8 h. The cells were treated with 3%, 6%, 9% or 12% (v/v) concentrations of ethanol, respectively. The optical density (OD) of the cultures was measured at 600 nm every 4 h. Each experiment was repeated three times.

For the survival analysis, *W. anomalus* cells were treated with 3%, 6%, 9%, or 12% (v/v) concentrations of ethanol for 6 h. Yeast cells were harvested, washed twice with distilled water, and re-suspended in distilled water at a same cell concentration (OD_600_ = 1). The cell suspension was diluted to 10^0^, 10^−2^, and 10^−4^, and 2 μL of each diluent was spotted onto YEPD solid plates. The cells were incubated at 28 °C for 36 h. Colonies were observed and captured using a microscope (Olympus, Japan).

The yeast cells under ethanol stress were monitored for 6 h, using methylene blue staining, and the death rate was calculated by counting the number of dead cells in a random location on the plate. Ten locations were observed for each sample.

Biomass was detected by measuring the dry weight of *W. anomalus* cells under different ethanol treatments. Briefly, 30 mL of *W. anomalus* cells were collected by centrifugation at the speed of 4, 000 × g for 10 min. The cells were dried at 65 °C until a constant weight was achieved, and the weight was measured using an analytical balance (Lichen, China).

### Transcriptomics analysis

*W. anomalus* cells were treated with 9% or 12% (v/v) concentrations of ethanol for 6 h, collected via centrifugation at the speed of 4, 000 × g for 10 min, frozen quickly using liquid nitrogen, and stored at − 80 °C for transcriptomics and metabolomics analyses.

Total RNA was extracted from the yeast cells using Trizol reagent (Invitrogen, USA) according to the manufacturer’s instructions, and genomic DNA was removed using DNase I (Takara, Japan). An RNA-seq transcriptome library was prepared using a TruSeqTM RNA sample preparation Kit from Illumina (San Diego, USA) and 1 μg of total RNA. Libraries were size-selected for cDNA target fragments of 300 bp on 2% Low Range Ultra Agarose, followed by PCR amplification using Phusion DNA polymerase (NEB, USA) for 15 PCR cycles. The paired-end RNA-seq sequencing library was sequenced with the Illumina HiSeq xten/NovaSeq 6000 sequencer (Illumina, USA).

The raw paired-end reads were trimmed and controlled by SeqPrep (https://github.com/jstjohn/SeqPrep) and Sickle (https://github.com/najoshi/sickle) with default parameters. Then, clean reads were separately aligned to reference genomes with orientation mode using HISAT2 (http://ccb.jhu.edu/software/hisat2/index.shtml) software. The mapped reads of each sample were assembled by StringTie (https://ccb.jhu.edu/software/stringtie/index.shtml? t = example) in a reference-based approach. To identify differentially expressed genes (DEGs), the expression level of each transcript was calculated according to the transcripts per million reads method. RSEM (http://deweylab.biostat.wisc.edu/rsem/) was used to quantify gene abundances.

In addition, KEGG pathway analysis was carried out by Goatools (https://github.com/tanghaibao/Goatools) and KOBAS (http://kobas.cbi.pku.edu.cn/home.do) [31–33].

### Non-targeted metabolomics

Liquid chromatography-mass spectrometry (LC–MS)-based metabolomics was conducted by Majorbio Biotech (Shanghai, China). Briefly, 50 mg of the ethanol-treated samples or control sample were accurately weighed, and the metabolites were extracted using 400 µL of methanol:water (4:1, v/v) solution, containing 0.02 mg/mL of L-2-chlorophenylalanine as the internal standard. The mixture was allowed to settle at − 20 °C, and then it was vortexed for 30 s and ultrasonicated at 40 kHz for 30 min at 5 °C. The supernatant was carefully prepared for LC–MS analysis after centrifugation at 13,000 × g for 15 min. The chromatographic separation and mass spectrometric conditions were from a previously published method by Chen [34]. The raw data were imported into Progenesis QI 2.3 (Waters, USA) for peak detection and alignment. The preprocessing results generated a data matrix comprised of retention time, mass-to-charge ratio values, and peak intensity. Annotation of the metabolites and differential metabolites between ethanol-treated groups and the control group was performed on the Majorbio Cloud Platform (https://cloud.majorbio.com).

### Real-time quantitative PCR (q-PCR) analysis

Total RNA was extracted from the ethanol-treated groups and control group using Trizol reagent (Sangon Biotech, China), according to the manufacturer's instructions. cDNA was transcribed using the PrimerScript RT Reagent Kit (Takara). Real-time PCR was performed using the Light Cycler 96 detection system (Roche, Germany). The primers used for q-PCR in this study are shown in Table S7. Data were normalized to the housekeeping gene *actin* and analyzed using the 2^−ΔΔCT^ method.  

### Growth analysis of W. anomalus by exogenous addition of amino acids

*W. anomalus* cells were cultured at 28 °C for 8 h and then divided into ten groups. For the control group and ethanol treatment groups, no ethanol and 9% or 12% (v/v) ethanol were added, respectively. For the amino acid supplementary groups, 9% or 12% (v/v) ethanol was added, along with 5 mM of exogenous aspartate, glutamate, or arginine. All the treatment groups were further cultured at 28 °C for 8 h. The cultures were diluted to 10^0^, 10^−2^, or 10^−4^, spread on YEPD solid medium and incubated at 28 °C for 36 h.

### Statistics

The results are expressed as the mean ± standard deviation. Univariate analysis of variance (ANOVA) of the data and the determination of the significance of the difference were performed using SPSS 21.0 software. *P* < 0.05 was considered statistically significant.

## Supplementary Information

**Additional file 1:** **FigureS1.** Cells death determination under different concentrations of ethanoltreatment by methylene blue staining. A,0% ethanol treatment group; B, 3% ethanol treatment group; C, 6% ethanoltreatment group; D, 9% ethanol treatment group; E, 12% ethanol treatment group.Bar=100 μm.**Figure S2.**  Results of principalcomponent analysis (PCA) of the samples for transcriptome sequencing. **FigureS3.**  PCA score plots of the samples for metabolomicsanalysis in positive and negative ion modes. A, Positive ion mode; B, Negativeion mode. **Table S7.** Primersused in this study for real-time quantitative PCR detection.

**Additional file 2:** **Table S1.**

**Additional file 3:** **Table S2.**

**Additional file 4:** **Table S3.**

**Additional file 5:** **Table S4.**

**Additional file 6:** **Table S5.**

**Additional file 7:** **Table S6.**

## Data Availability

All data generated or analyzed during this study are included in this published article and its supplementary information files.

## Abbreviations

W. anomalus: Wickerhamomyces anomalus
K. marxianus: Kluyveromyces marxianus
S. cerevisiae: Saccharomyces cerevisiae
DEGs: Differentially expressed genes
DEMs: Differentially expressed metabolites
TCA cycle: Tricarboxylic acid cycle
RNA-seq: RNA-sequencing
VIP: Variable importance in the projection
PBS: Phosphatic buffer solution
OD: Optical density
q-PCR: Quantitative PCR
