# Supplementary material for: Analysis of the ethanol stress response mechanism in Wickerhamomyces anomalus based on transcriptomics and metabolomics approaches
Source: BMC Microbiol. 2022 Nov 15;22:275. doi: 10.1186/s12866-022-02691-y (PMC9664796; doi:10.1186/s12866-022-02691-y)
Supplement: Supplementary file 1 — Additional file 1: FigureS1. Cells death determination under different concentrations of ethanoltreatment by methylene blue staining. A,0% ethanol treatment group; B, 3% ethanol treatment group; C, 6% ethanoltreatment group; D, 9% ethanol treatment group; E, 12% ethanol treatment group.Bar=100 μm.Figure S2. Results of principalcomponent analysis (PCA) of the samples for transcriptome sequencing. FigureS3. PCA score plots of the samples for metabolomicsanalysis in positive and negative ion modes. A, Positive ion mode; B, Negativeion mode. Table S7. Primersused in this study for real-time quantitative PCR detection. [file 12866_2022_2691_MOESM1_ESM.docx]

Figure S1. Cells death determination under different concentrations of ethanol treatment by methylene blue staining. A, 0% ethanol treatment group; B, 3% ethanol treatment group; C, 6% ethanol treatment group; D, 9% ethanol treatment group; E, 12% ethanol treatment group. Bar=100 μm.


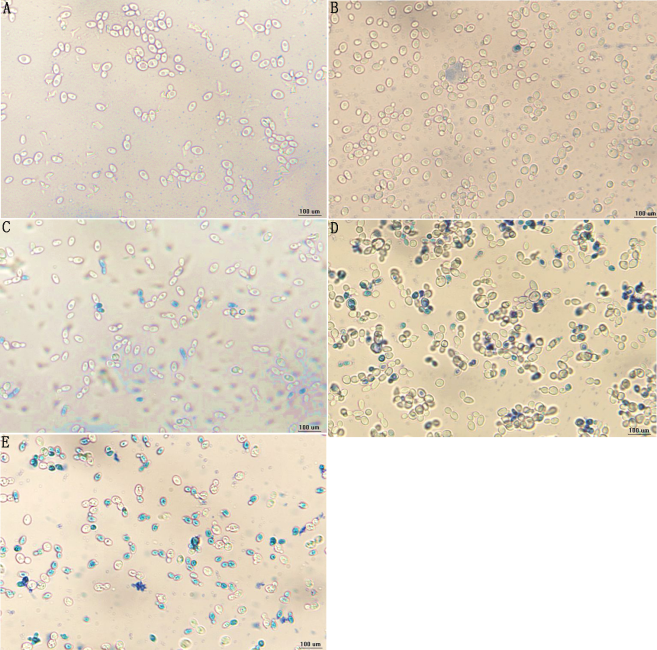


Figure S2. Results of principal component analysis (PCA) of the samples for transcriptome sequencing.


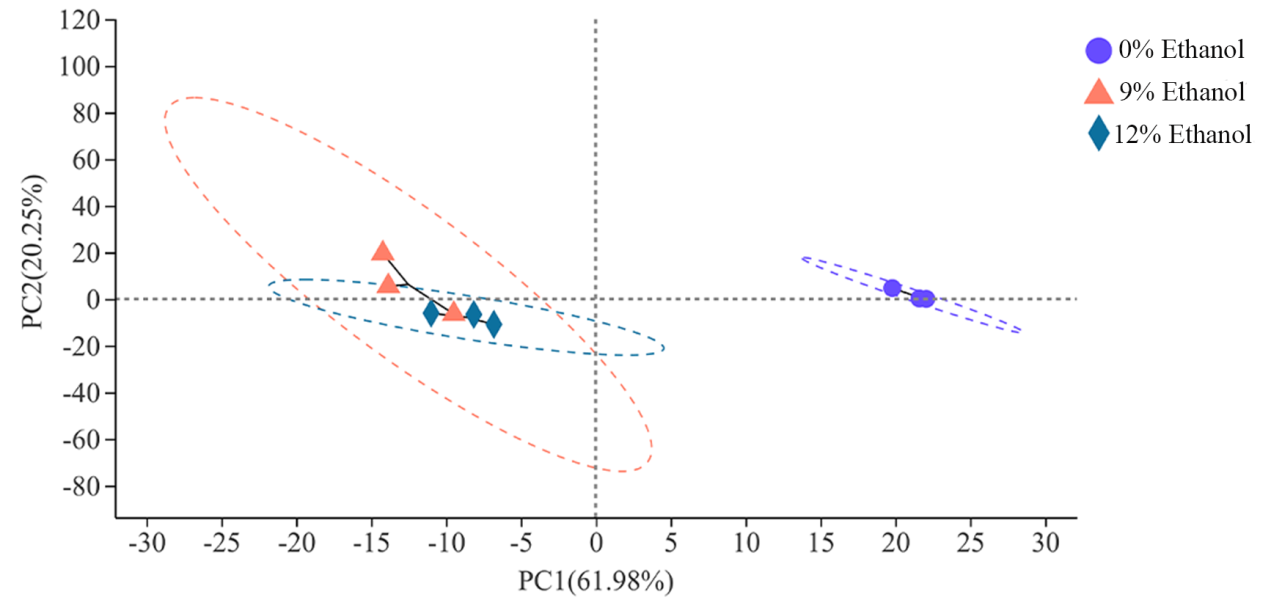


Figure S3. PCA score plots of the samples for metabolomics analysis in positive and negative ion modes. A, Positive ion mode; B, Negative ion mode.


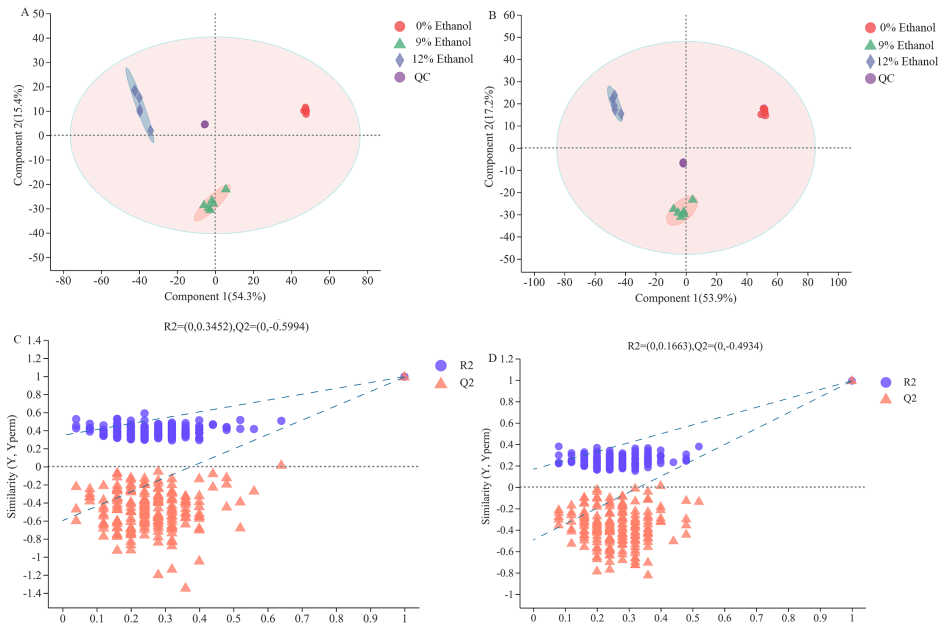


Table S7. Primers used in this study for real-time quantitative PCR detection.

| Number | Primer name | Sequences (5’-3’) | Product size (bp) |
| --- | --- | --- | --- |
| 1 | *Wa60613*-F | CCGTGATGTTGTGGATGCTG | 75 |
| 2 | *Wa60613*-R | ACCAGCTAACCCACCACTTG |  |
| 3 | *Wa78611*-F | CGTCGATGTCGCTACTGTCT | 267 |
| 4 | *Wa78611*-R | GCAGCTTCACCGTGTTCTTG |  |
| 5 | *Wa13980*-F | TCACAGATTGCAACTTTAGCTAGT | 90 |
| 6 | *Wa13980*-R | TAGCCCATCAGCTGTGAAAG |  |
| 7 | *Wa63188*-F | ACTTGGGGTCCAGTTGCTTT | 86 |
| 8 | *Wa63188*-R | GCAGCAGTAGCAAGACCCAT |  |
| 9 | *Wa46076*-F | CACATCTCCAGCTTCCCCAG | 230 |
| 10 | *Wa46076*-R | CTTCTTCAGCACCAGCAACG |  |
| 11 | *Wa26420*-F | TGATGCTTATGCTGGTTGGGA | 160 |
| 12 | *Wa26420*-R | ACCAGCATTCCAGATGGTGA |  |
| 13 | *Wa-60885*-F | AGGGGCCATCTCATTCTGGA | 172 |
| 14 | *Wa60885*-R | ACCAGCTAACCCACCACTTG |  |
| 15 | *Wa34481*-F | CCAAAACTTCCCAGGCTTGC | 74 |
| 16 | *Wa34481*-R | TAGCAGGCCATGAACCAGTG |  |
| 17 | *Wa92106*-F | ACTCTTCATCCCCATCAATCAACA | 82 |
| 18 | *Wa92106*-R | GATGGAGGACCATTGGAAGCA |  |
| 19 | *Wa31465*-F | AGGTGCAGGTCGTGAAGATG | 219 |
| 20 | *Wa31465*-R | TCACGACGTCCCAAAGATGG |  |
| 21 | *Waactin*-F | GGTACCACCATGTTCCCAGG | 102 |
| 22 | *Waactin*-R | ACGTTCTGGTGGAGCAATGA |  |
